# Climate change and the different roles of physicians: a critical response to "A Planetary Health Pledge for Health Professionals in the Anthropocene"

**DOI:** 10.1007/s11019-021-10051-2

**Published:** 2021-09-16

**Authors:** Urban Wiesing

**Affiliations:** Institute for Ethics and History of Medicine, University of Tübingen, Gartenstr. 47, 72074 Tübingen, Germany

**Keywords:** Declaration of Geneva, Planetary Health Pledge, Climate change, Professionalism

## Abstract

The article critically responds to "A Planetary Health Pledge for Health Professionals in the Anthropocene" which was published by Wabnitz et al. in *The Lancet* in November 2020. It focuses on the different roles and responsibilities of a physician. The pledge is criticised because it neglects the different roles, gives no answers in case of conflicting goals, and contains numerous inconsistencies. The relationship between the Planetary Health Pledge and the Declaration of Geneva is examined. It is argued that the Planetary Health Pledge should have supplemented the Declaration of Geneva instead of changing it.

There is no doubt that medicine and climate change are closely linked. On the one hand the current climate change has a massive impact on the individual's health (Song et al. 2017; Watts et al. 2021). On the other hand, medicine produces greenhouse gases and thus contributes to climate change (Health Care without Harm). A wide literature has addressed these issues and made proposals for needed reactions to this challenge. Some authors are convinced that new ethical rules must become visible in a pledge for health professionals. Therefore, in November 2020, "A Planetary Health Pledge for Health Professionals in the Anthropocene" was published in *The Lancet* (Wabnitz et al. 2020). In light of the dramatic environmental changes, the authors want to define a new ethical basis for healthcare professionals and to “transform human values, behaviours, and societal structures” (Wabnitz et al. 2020, p. 1472). Here, critical remarks should be raised, especially from the perspective of professionalism. To avoid misunderstandings: the current climate change is a disaster of the utmost extent for humanity. Medical professions have the knowledge and skills to research and mitigate this impact. They need to contribute their knowledge and skills to the societal debate. But what does this mean for a healthcare professional?

## The different roles of physicians

Physicians, as well as other healthcare professionals, are active in various roles. They can be involved in the physician–patient relationship, but they can also act as consultants, as public health experts, or as experts in political institutions or public discussions. The most important medical role is the one towards a patient. In this relationship, a physician is to a large extent relieved of further social responsibilities and should act primarily in a way that is in the patient’s best interest. The same is true for other healthcare professionals, but interestingly also for priests and lawyers, when they are in professional contact with individual believers or defendants. They also must focus on the individual may it be a patient, believer, or client. If conflicts were to arise with other roles of the physician, the role towards a patient mostly has precedence.

The Declaration of Geneva (DoG) of the World Medical Association confirms this, with the statement: “The health and well-being of my patient will always be my first consideration.” (World Medical Association 2017).

This is underlined by the individual orientation of the whole document. The DoG is written entirely in the singular and always speaks in terms of “the patient” and “the physician.” It primarily addresses the duties in an individual physician–patient relationship. The World Medical Association strongly believes that this is the primary responsibility of a physician. First and foremost, a physician is obligated to an individual patient and his or her health.

In this respect, it is surprising that the Planetary Health Pledge (PHP) does not refer to a patient in the singular, but rather always refers to “people,” “persons,” or “patients” in the plural form. Why should the individualistic orientation of the medical profession change? Why does the accompanying text to the PHP not address this alteration? Historical experiences, where physicians were primarily obligated to a group (be that race, class, or nationality) should have cautioned the authors of the PHP. To put it clearly, the authors of the PHP should not be accused of having similar intentions as totalitarian regimes; their intention is convincing and worthy of support. But there is a parallel: In both cases, the individual responsibility in the doctor-patient relationship is extended by a collective one. But in his or her central role it is not the first task of a physician to improve the health of a group. Rather a physician should do what is best for the individual patient in the physician–patient relationship—which might as a result improve the health of a group. Conflicts can arise between an individual orientation in the physician–patient relationship and political guidelines, even in environmental protection.

According to a study by "Health Care without Harm", the health sector contributes 4.4% to global emissions (Health Care without Harm). In terms of climate protection these emissions must be reduced, also for the sake of health. But should physicians in the physician–patient relationship therefore refrain from taking helpful measures? Environmental protection and medical care can lead to conflicting goals. Changing a medical procedure to protect the environment may result in the patient being better off in case of unnecessary treatment or the medical outcome being just as good. These two cases are unproblematic, the first case is even desirable for two reasons. However, if the best intervention is not chosen for environmental reasons and the patient is not treated optimally and worse off, then there is a serious conflict. This is exactly what the PHP ignores. How should a physician act when a medically required procedure for a patient imposes a significant environmental burden? Should the physician withhold the treatment because it produces copious amounts of CO_2_? What should be done when adequate individual medicine conflicts with the requirements of environmental protection? How much harm towards patients is justifiable for the good of the environment? And for the integrity of the profession, the central question arises: who should decide this? Should a physician weigh at the bedside a concrete suffering of a patient against the protection of the environment? Who gives him the authority to do this and where does he get the concrete benchmarks?

Especially the responsibilities in environmental protection are difficult to define. The polluters are often not confronted with the consequences and the results of the environmental changes show up only in later generations. It is difficult to determine exactly how much an individual action contributes to environmental harm (see e.g. Wardrope 2020). In view of this complexity, it is even more important to establish the clear responsibility in the different roles of a doctor. As long as physicians have two equally helpful measures, they can be expected to choose the less environmentally harmful measure. But should physicians have to decide personally in the physician–patient relationship whether to help a patient or reduce greenhouse gas emissions? No, physicians must be relieved of such decisions in the physician–patient relationship, otherwise trust in the medical profession will be lost. These are decisions that need to be clarified at the societal level. The PHP fails to address this conflict and to describe different responsibilities. It leaves doctors alone when they are confronted with conflicting goals in the physician–patient-relationship.

## No obligation to confidentiality and no ban on discrimination?

What do “planetary health values” (Wabnitz et al. 2020, p. 1472) mean for the obligation to confidentiality? This is not touched upon in the PHP, although it is a central obligation of the DoG. If a physician discovers within the course of treatment, that a patient is violating environmental protection regulations and intends to do so in the future, should the physician report this to the police or environmental authorities? Thus far, confidentiality prohibits such behavior for a good reason mentioned above: trust. The PHP correctly states: “Health professionals are among the most trusted members of society.” (Wabnitz et al. 2020, p. 1472). In part, they are trusted because there is an obligation to confidentiality. But why is the obligation to confidentiality missing from the PHP? Should it be invalidated when a patient harms the environment?

The DoG details criteria that do not influence the individual physician–patient relationship: “I will not permit considerations of age, disease or disability, creed, ethnic origin, gender, nationality, political affiliation, race, sexual orientation, social standing or any other factor to intervene between my duty and my patient.” Why does the PHP not adopt this paragraph? The explanatory text to the PHP mentions the adverse health consequences of “any discrimination, including that involving gender, race and ethnicity” (Wabnitz et al. 2020, p. 1472). But it does not state that these criteria may not affect the physician–patient relationship. And do the other criteria of the DoG (age, disease or disability, creed, nationality, political affiliation, sexual orientation, or social standing) become irrelevant when guided by the “planetary health values” (Wabnitz et al. 2020, p. 1472)?

The move away from the individualistic orientation of the medical profession can also be seen in the PHP in the prevention of disinformation. It prescribes: A physician should challenge “attempts at spreading disinformation that can undermine planetary health.” Why does the PHP not mention disinformation that can undermine the health of a patient? In paragraph nine, the PHP states: “I will share and expand my knowledge for the benefit of society and the planet.” But the DoG states “for the benefit of the patient and the advancement of healthcare.” Why did the authors of the PHP take this out? Are the benefits of the individual patient irrelevant when sharing knowledge?

## A new role for physicians?

All of these questions raised by the PHP point to a role change for physicians. With this, the PHP contrasts other codifications of a physician’s professionalism that in particular emphasize individual focus. For example, the project “The Medical Professionalism in the New Millennium: A Physician Charter 2002”, declared patient’s welfare to be the primary principle of professionalism: “Principle of primacy of patient welfare. This principle is based on a dedication to serving the interest of the patient. […] Market forces, societal pressures, and administrative exigencies must not compromise this principle.” (ABIM 2002) What should happen if environmental restrictions “compromise this principle”? And what does this mean for physician–patient relationships? These questions remain unanswered in the PHP.

One of the sentences of the DoG is as follows. “I will respect the autonomy and dignity of my patient”. The PHP changes this sentence significantly and adds to autonomy and dignity the principle of non-maleficence and a new approach. “To do no harm, I will respect the autonomy and dignity of all persons in adopting an approach to maintaining and creating health which focuses on prevention of harm to people and planet”. This sentence names the three principles in such a way that they would easily complement each other. But this is a romantic idealization. It is well known that these principles can come into conflict with each other and may be difficult to operationalise in practice (Beauchamp and Childress 2019). To respect the autonomy of the patient neither automatically means to prevent harm to people and planet nor to focus on prevention. To adopt the new approach may easily come into conflict with the respect of autonomy “of all persons”.

Furthermore, numerous statements within the PHP raise questions. In the DoG, a physician commits themselves to a practice according to “good medical practice.” In the PHP, health care professionals only commit themselves to “good practice.” Why was the term “medical” omitted? This should at least be explained in the accompanying article—but it is not. In the twelfth paragraph, the PHP speaks of “the human right to health”. However, this is not the case in the DoG and is at the very least misleading. Health is not a human right, the right to health*care* can be seen as a human right.

## Who is responsible for healthcare policy decisions?

The authors also mention the COVID 19 pandemic and see it as an argument for their PHP article. Conversely, the COVID 19 pandemic also reveals the need to distinguish between the different roles of a physician. It clearly shows that important ethical decisions, which are related to society, must be made outside of the physician–patient relationship. Criteria for triage of ICU beds should not be determined by a single physician but should rather be determined outside of the physician–patient relationship with social legitimation. Similarly, the prioritization of vaccination, which has a major impact on the health of a society, should not be decided by an individual physician. As experts, doctors should contribute their knowledge and experience to the public. But society must set the criteria by which ICU beds or vaccinations are to be allocated. The different levels at which decisions must be made in healthcare are withheld in the PHP. The accompanying article does not illuminate such conflicts, which may also exist between individual medical practice and environmental protection. The responsibilities that are linked to the different roles of a physician are muddled by the PHP.

## Supplementing or changing the Declaration of Geneva?

The Declaration of Geneva focuses almost exclusively on the role of a doctor towards his or her patient. It regulates only the physician–patient-relationship and does not address the ethical behaviour as consultants, as public health experts, or as experts in political institutions. It may therefore make sense to supplement the DoG, also with reference to environmental protection. But what does the PHP do? It explicitly borrows from the Geneva Pledge; it “is based on the Declaration of Geneva” (Wabnitz et al. 2020, p. 1473). Nine out of 13 paragraphs address a topic that is also found in the DoG, using sub-sentences and certain terms of the DoG verbatim. Therefore, the PHP not only supplements the Declaration of Geneva, but modifies it fundamentally, even when the PHP is not concerned with environmental protection. Are the changes of the DoG made by the PHP justified and convincing? Neither! The changes of the moral orientation of the DoG are not even explained. They lead to results that are critical for the patient–physician-relationship and the trust in medicine. For the medical profession it would be dangerous to fundamentally change the moral core of the DoG. Instead of changing the ethics of the DoG it would have been more convincing to supplement it.

## Conclusion

The intentions of the PHP are commendable, but the document does not address a fundamental problem and leaves many questions unanswered. It alters the central role of the physician and ignores conflicting goals that can arise between a physician’s role in the physician–patient relationship and environmental protection. In their roles outside of the physician–patient relationship, physicians are supposed to advocate for environmental protection. In their relationship with a patient, however, they are supposed to be concerned with the patient’s well-being. Moreover, the PHP contains many inconsistencies and keeps quiet about key obligations of a physician. It leaves doctors alone at the bedside.
